# Inflammatory and Oxidative Stress Biomarkers in Gingival Crevicular Fluid (GCF) and Serum of Dogs With Gingivitis and Periodontitis

**DOI:** 10.1155/ijod/4652796

**Published:** 2026-07-23

**Authors:** Doğu Turgut, Efe Kurtdede

**Affiliations:** ^1^ Institute of Health Sciences, Veteriner Biyokimya Anabilim Dalı, Ankara University, Dışkapı, Ankara, Türkiye, ankara.edu.tr; ^2^ Faculty of Veterinary Medicine, Department of Biochemistry, Ankara University, Dışkapı, Ankara, Türkiye, ankara.edu.tr

**Keywords:** canine gingivitis, canine periodontitis, gingival crevicular fluid, neopterin, oxidative stress

## Abstract

**Introduction/Objectives:**

Periodontal disease is one of the most common oral disorders in dogs and is characterized by inflammation and destruction of periodontal tissues. This study aimed to compare local and systemic inflammatory and oxidative stress biomarkers in dogs with gingivitis and periodontitis and to evaluate neopterin as a potential biomarker of periodontal inflammation.

**Materials and Methods:**

A total of 52 dogs were included, comprising 26 dogs with gingivitis and 26 dogs with periodontitis. Gingival crevicular fluid (GCF), blood, and serum samples were collected. Total oxidant status (TOS), total antioxidant status (TAS), caveolin‐1, monocyte chemoattractant protein‐1 (MCP‐1), interleukin (IL)‐1β, IL‐6, neopterin, and selected hematological variables were measured. Biomarker concentrations and correlations between serum and GCF were evaluated.

**Results:**

Dogs with periodontitis exhibited significantly higher serum TOS, TAS, and IL‐6 concentrations than dogs with gingivitis (*p*  < 0.001). In contrast, serum and GCF concentrations of IL‐1β, MCP‐1, caveolin‐1, and neopterin were significantly higher in dogs with gingivitis (*p*  < 0.001). Neutrophil and monocyte counts were also higher in the gingivitis group. Significant correlations were identified between serum and GCF biomarker concentrations.

**Conclusions:**

Periodontal disease in dogs was associated with distinct stage‐dependent inflammatory and oxidative stress profiles. Gingivitis was characterized by increased levels of several inflammatory mediators, whereas periodontitis was associated with increased oxidative stress and IL‐6 concentrations. These findings suggest that local and systemic biomarker responses vary according to periodontal disease stage and warrant further investigation of GCF biomarkers for monitoring periodontal inflammation.

## 1. Introduction

Periodontal disease is among the most prevalent oral inflammatory conditions in dogs. The disease is initiated by the accumulation of a bacterial dental plaque biofilm within the gingival sulcus, where it comes into direct contact with host tissues and triggers a host‐mediated inflammatory response. Importantly, supragingival plaque located on intact enamel surfaces, particularly on cusp tips, does not directly induce periodontal tissue inflammation unless it extends into the gingival sulcus [1–3].

Gingivitis is the earliest clinically detectable stage of periodontal disease and represents a reversible inflammatory condition confined to the gingival tissues without the loss of periodontal attachment. Clinically, gingivitis may present in varying degrees of severity, ranging from mild gingival erythema to moderate or severe inflammation characterized by edema, bleeding on probing, and increased gingival sensitivity. In all stages, however, the defining feature of gingivitis is the absence of attachment loss and alveolar bone destruction. If untreated, gingivitis may progress to periodontitis, an irreversible condition characterized by periodontal attachment loss, periodontal pocket formation, and alveolar bone resorption [1, 3].

The progression from gingivitis to periodontitis is not solely dependent on the bacterial load but is strongly influenced by the host immune‐inflammatory response [4, 5]. Dysregulated activation of innate and adaptive immunity leads to excessive production of cytokines, chemokines, and reactive oxygen species (ROS), contributing to connective tissue breakdown and bone resorption [4, 6].

Importantly, periodontal inflammation is not confined to the oral cavity. Locally produced inflammatory mediators may enter the systemic circulation, resulting in measurable systemic inflammatory and oxidative stress responses. This has led to increasing interest in periodontal disease as a potential contributor to the systemic inflammatory burden in dogs [6, 7].

Gingival crevicular fluid (GCF) reflects the local inflammatory microenvironment of periodontal tissues, while serum biomarkers reflect systemic involvement. Among these, neopterin is a marker of macrophage activation and cellular immune response, while interleukin (IL)‐1β and IL‐6 are key proinflammatory cytokines involved in periodontal tissue destruction [8–10].

Despite the growing interest, most available studies evaluate either local or systemic biomarkers separately. The simultaneous assessment of both compartments remains limited, particularly in veterinary dentistry, where disease staging may influence biomarker expression patterns. Moreover, periodontal inflammation is increasingly recognized as a dynamic process in which biomarker expression may vary across disease stages rather than increasing in a strictly linear fashion. Therefore, comparative evaluation between gingivitis and periodontitis may provide additional insights into stage‐specific immune responses.

## 2. Materials and Methods

### 2.1. Ethical Approval

This study was approved by the Ankara University Local Ethics Committee on Animal Experiments (Decision Number 2024‐28‐154).

### 2.2. Animal Material and Study Groups

A total of 52 client‐owned dogs aged ≥3 years were included in the study. The study population consisted predominantly of small‐ and toy‐breed dogs, including chihuahua, pekingese, pomeranian, toy or miniature poodle, and yorkshire terrier.

Dogs were eligible for inclusion if they exhibited clinical signs of periodontal disease, had undergone a complete periodontal examination and intraoral radiographic evaluation, and had not received periodontal treatment within the previous 6 months. Dogs with systemic inflammatory disease, endocrine disorders, neoplasia, renal or hepatic insufficiency, pregnancy, or a history of antimicrobial, corticosteroid, or non‐steroidal anti‐inflammatory drug use within 4 weeks prior to sampling were excluded.

Information regarding diet and oral hygiene practices was obtained from the owners. All dogs were reportedly fed commercially prepared dry food, whereas oral hygiene measures were inconsistently applied.

### 2.3. Group Classification

Dogs were classified into two groups based on full‐mouth periodontal examination under general anesthesia, including standardized periodontal probing and intraoral radiography. Periodontal probing depth measurements were obtained from multiple sites around each tooth, and full‐mouth dental radiographs were acquired to assess the periodontal attachment and alveolar bone status.

Gingivitis was defined as the presence of gingival inflammation (erythema, edema, and/or bleeding on probing) without clinical attachment loss or radiographic evidence of alveolar bone loss, with periodontal probing depths ≤3 mm. Gingivitis severity was not further subclassified because the objective was to compare biomarker profiles between disease stages rather than among severity categories within a single stage.

Periodontitis was defined as the presence of clinical attachment loss, periodontal pocketing (>3 mm), gingival recession, and radiographic evidence of alveolar bone loss.

Although periodontitis severity may be further subclassified according to attachment loss and radiographic findings, severity staging was not performed in the present study because the primary objective was to compare inflammatory and oxidative biomarkers between gingivitis and periodontitis groups.

### 2.4. Blood and Serum Collection

Blood samples were collected from the cephalic vein into EDTA and plain collection tubes. Hematological analyses were performed using an automated hematology analyzer within 1 h after the collection. Serum samples were separated by centrifugation and stored at −80°C until the analysis.

### 2.5. GCF Sampling

GCF samples were collected from the distobuccal aspect of the maxillary fourth premolar tooth (tooth 108/208), selected due to its high susceptibility to periodontal disease in dogs and its suitability for standardized sampling across individuals.

In each dog, the sampled tooth met the diagnostic criteria for the assigned periodontal group. In the gingivitis group, the tooth exhibited gingival inflammation without attachment loss or radiographic bone loss. In the periodontitis group, the tooth showed clinical attachment loss and radiographic evidence of alveolar bone loss.

All samples were immediately diluted with cold phosphate‐buffered saline (PBS) and centrifuged at 13,000 × *g* for 20 min at 4°C, and the supernatants were stored at −80°C until analysis.

PBS lavage was used to obtain a sufficient sample volume for multiplex biomarker analysis. Although this method may dilute absolute biomarker concentrations, all samples were processed using identical protocols across both groups. Therefore, internal comparisons between groups were considered appropriate. However, absolute biomarker concentrations and comparisons with studies using absorbent paper strip sampling methods should be interpreted with caution. Despite this limitation, PBS lavage has previously been used in veterinary periodontal biomarker studies when larger sample volumes are required.

### 2.6. Biochemical and Immunological Analyses

Oxidative stress status was evaluated by measuring total oxidant status (TOS) and total antioxidant status (TAS) using commercial spectrophotometric kits (Rel Assay Diagnostics, Gaziantep, Türkiye; TOS: Cat. No. RL0024, TAS: Cat. No. RL0017).

Serum and GCF levels of caveolin‐1, monocyte chemoattractant protein‐1 (MCP‐1), IL‐1β, IL‐6, and neopterin were measured using commercial canine ELISA kits according to the manufacturers’ instructions: neopterin (BT LAB, Shanghai, China; Cat No. E0205Ca) and MCP‐1 (BT LAB, Shanghai, China; Cat No. E0135Ca), IL‐1β (BT LAB, Shanghai, China; Cat. No. E0002Ca), IL‐6 (BT LAB, Shanghai, China; Cat No. E0004Ca) and caveolin‐1 (BT LAB, Shanghai, China; Cat No. E0012Ca).

Serum biochemical analyses were performed using a Mindray BS120 Biochemistry Analyzer (Mindray Bio‐Medical Electronics Co., China). Hematological parameters were analyzed in EDTA blood samples using a Mindray BC‐5000 hematology analyzer (Mindray Bio‐Medical Electronics Co., China).

### 2.7. Statistical Analysis

Statistical analyses were performed using Stata 12/MP4 software. The normality of data distribution was assessed using the Shapiro–Wilk test. Homogeneity of variances was evaluated using Levene’s test. Data are presented as the mean ± standard deviation (SD).

Comparisons between groups were performed using the independent sample *t*‐test for normally distributed variables and the Mann–Whitney *U* test for nonnormally distributed variables. Correlations between serum and GCF biomarkers were analyzed using Spearman’s correlation analysis. *p*‐Values <0.05 were considered statistically significant.

## 3. Results

Dogs with periodontitis were characterized by gingival recession, dental calculus accumulation, halitosis, bleeding on probing, periodontal pocket formation, and radiographic evidence of alveolar bone loss.

Serum biochemical and immunological findings are presented in Table 1.

**Table 1:** Levels of biochemical and immunological serum variables in dogs with gingivitis and periodontitis.

Variable	Gingivitis (mean ± SD)	Periodontitis (mean ± SD)	*p*‐Value
Total oxidant status (TOS, μmol/L)	2.34 ± 0.40	3.95 ± 0.89	<0.001
Total antioxidant status (TAS, mmol/L)	17.54 ± 1.11	21.49 ± 0.89	<0.001
Caveolin‐1 (pg/mL)	1524.04 ± 148.86	704.81 ± 101.18	<0.001
Monocyte chemoattractant protein‐1 (MCP‐1, pg/mL)	285.69 ± 15.92	88.98 ± 18.94	<0.001
Interleukin‐1β (IL‐1β, pg/mL)	15.24 ± 4.07	2.74 ± 0.58	<0.001
Neopterin (nmol/L)	6.31 ± 1.97	1.77 ± 0.51	<0.001
Interleukin‐6 (IL‐6, pg/mL)	2.46 ± 0.50	3.95 ± 0.89	<0.001
Aspartate aminotransferase (AST, U/L)	39.23 ± 6.03	40.66 ± 6.17	0.402
Alanine aminotransferase (ALT, U/L)	57.75 ± 11.81	56.11 ± 11.56	0.615
Total protein (TP, g/dL)	9.83 ± 1.25	9.81 ± 1.43	0.971
Albumin (ALB, g/dL)	4.81 ± 0.44	4.62 ± 0.41	0.111
C‐reactive protein (CRP, mg/L)	11.52 ± 2.59	11.58 ± 2.60	0.855

*Note*: Data are presented as mean ± standard deviation (SD). Statistical significance was set at *p* < 0.05. Significant differences were identified between groups in TOS, TAS, caveolin‐1, MCP‐1, IL‐1β, neopterin, and IL‐6 (*p*  < 0.001). Biomarker profiles showed distinct stage‐dependent patterns, with higher IL‐6 and oxidative stress markers (TOS and TAS) in periodontitis, whereas IL‐1β, MCP‐1, caveolin‐1, and neopterin were increased in gingivitis. No significant differences were detected for AST, ALT, total protein, albumin, or CRP.

Hematological findings are shown in Table 2.

**Table 2:** Hematological parameters in dogs with gingivitis and periodontitis.

Variable	Gingivitis (mean ± SD)	Periodontitis (mean ± SD)	*p*‐Value
White blood cells (WBC, × 10^3^/μL)	9.24 ± 1.53	9.41 ± 0.73	0.615
Neutrophils (× 10^3^/μL)	6.97 ± 0.83	5.94 ± 0.92	<0.001
Lymphocytes (× 10^3^/μL)	3.65 ± 0.72	3.54 ± 0.67	0.577
Monocytes (× 10^3^/μL)	2.44 ± 0.18	2.32 ± 0.21	0.002

*Note*: Values are presented as mean ± standard deviation (SD), and *p*‐values were calculated using appropriate statistical tests. *p* < 0.05 was considered statistically significant. Neutrophil and monocyte counts were significantly higher in the gingivitis group, while no differences were observed for total leukocyte or lymphocyte counts.

GCF findings are presented in Table 3.

**Table 3:** Levels of GCF variables in dogs with gingivitis and periodontitis.

GCF variable	Gingivitis (mean ± SD)	Periodontitis (mean ± SD)	*p*‐Value
Caveolin‐1 (pg/mL)	410.93 ± 67.37	172.63 ± 27.54	<0.001
MCP‐1 (pg/mL)	2.52 ± 0.50	1.58 ± 0.23	<0.001
IL‐1β (pg/mL)	5.04 ± 0.87	2.00 ± 0.37	<0.001
Neopterin (nmol/L)	5.00 ± 1.19	1.62 ± 0.45	<0.001
IL‐6 (pg/mL)	1.65 ± 0.23	3.25 ± 0.39	<0.001

*Note*: Data are presented as mean ± standard deviation (SD). Caveolin‐1, MCP‐1, IL‐1β, and neopterin levels were higher in the gingivitis group, whereas IL‐6 levels were higher in the periodontitis group (*p* < 0.001).

Abbreviation: GCF, gingival crevicular fluid.

The correlation analysis results are shown in Table 4.

**Table 4:** Correlation between serum and GCF levels of study variables.

Serum variable	GCF variable	Spearman’s correlation coefficient (*r*_*s*_)	*p*‐Value
Caveolin‐1	Caveolin‐1	−0.65	<0.001
MCP‐1	MCP‐1	−0.68	<0.001
IL‐1β	IL‐1β	0.83	<0.001
Neopterin	Neopterin	0.82	<0.001
IL‐6	IL‐6	0.87	<0.001

*Note*: Significant correlations were observed between serum and corresponding GCF levels of all evaluated biomarkers. *r*_*s*_, Spearman’s correlation coefficient.

Abbreviation: GCF, gingival crevicular fluid.

## 4. Discussion

The present findings highlight a stage‐dependent variation in inflammatory and oxidative stress responses in canine periodontal disease, suggesting a nonlinear disease‐associated biomarker profile. Notably, biomarker patterns did not show a uniform increase with disease severity, suggesting a nonlinear, stage‐dependent immune response consistent with previously described episodic progression models of periodontal disease [11–13].

Gingivitis represents a reversible inflammatory process limited to gingival tissues, whereas periodontitis is characterized by irreversible destruction of the periodontal supporting structures [1–3].

In veterinary dentistry, diagnosis primarily relies on clinical examination, periodontal probing, and radiographic assessment. However, these methods largely reflect structural changes rather than the underlying inflammatory activity. Therefore, evaluation of biomarkers in GCF and serum may provide additional information regarding disease activity [1, 2]. Gingivitis and periodontitis are increasingly recognized as distinct inflammatory states. Although periodontitis may develop from unresolved gingival inflammation in susceptible individuals, disease progression does not necessarily follow a strictly linear continuum [4, 11]. Periodontal disease progression is characterized by episodic bursts of inflammatory activity interspersed with periods of quiescence rather than continuous linear deterioration [12, 13]. Consequently, host immune‐inflammatory and biomarker responses may show stage‐dependent patterns that are not always proportional to clinical severity, indicating a complex nonlinear disease process [4, 6]. Instead of a uniform increase in biomarkers with disease severity, distinct stage‐dependent patterns were observed, supporting the concept that gingivitis and periodontitis represent biologically distinct inflammatory states rather than a simple linear continuum. Overall, these findings support the concept that periodontal disease is a host‐mediated condition characterized by dynamic immune‐inflammatory regulation rather than a uniform increase in inflammatory mediators with disease severity [1–5].

The present study further demonstrates that canine periodontal disease is associated with distinct stage‐dependent alterations in local (GCF) and systemic (serum) inflammatory and oxidative stress biomarkers. IL‐6 and TOS were significantly higher in dogs with periodontitis, suggesting sustained systemic inflammation and increased oxidative stress in advanced diseases. IL‐6 is a key cytokine involved in chronic inflammatory amplification and systemic immune modulation in periodontal disease [14], supporting its role in persistent and systemic disease activity. In contrast, IL‐1β, MCP‐1, neopterin, and caveolin‐1 were higher in the gingivitis group, which may reflect early‐stage immune activation or stage‐dependent differences in inflammatory dynamics. This pattern may reflect differences in immune response dynamics between disease stages [15, 16].

Gingivitis is generally associated with an early inflammatory response characterized by active leukocyte recruitment, whereas periodontitis is more closely associated with chronic inflammation and progressive tissue destruction, although overlap between stages may occur [4, 6, 17, 18]. IL‐1β is a key inflammatory mediator reflecting host immune activation, although it has also been associated with periodontal disease severity in previous studies [4]. Similarly, MCP‐1, neopterin, and caveolin‐1 may participate in early immune cell signaling and localized inflammatory processes, although their stage‐specific roles in canine periodontal disease remain unclear. In chronic periodontal disease, persistent inflammation may shift toward tissue remodeling and oxidative stress‐related pathways, potentially contributing to altered circulating levels of these mediators in advanced stages [15, 16]. Higher neutrophil and monocyte counts observed in the gingivitis group are consistent with early systemic immune activation and active leukocyte mobilization. In contrast, chronic periodontitis may involve altered leukocyte trafficking and redistribution between peripheral circulation and periodontal tissues, potentially explaining differences in circulating immune cell counts [19, 20].

The increased TAS observed in periodontitis may represent a compensatory response to sustained oxidative stress, although antioxidant responses in chronic inflammatory conditions are often variable. Chronic inflammatory conditions may induce adaptive upregulation of endogenous antioxidant defenses in response to the prolonged oxidative stress burden [7, 8].

Significant correlations were observed between serum and GCF levels of all evaluated biomarkers, including MCP‐1, neopterin, caveolin‐1, IL‐1β, and IL‐6, suggesting a link between local periodontal inflammation and systemic inflammatory status [21–26]. These findings are consistent with previous reports indicating that periodontal inflammation may be reflected systemically through circulating mediators. The observed correlations (*r*_*s*_ = 0.65–0.87) indicate moderate‐to‐strong associations between local and systemic inflammatory activity. However, these associations likely reflect shared inflammatory pathways rather than direct causality.

A major limitation of this study is the absence of a healthy control group; therefore, results should be interpreted as interstage differences rather than deviations from physiological baseline values [1–3]. Additionally, GCF was collected using PBS lavage, which may dilute absolute biomarker concentrations and limits direct comparison with studies using absorbent strip methods. However, identical processing across all samples minimizes potential internal bias.

Another limitation is the lack of severity stratification within the periodontitis group, which may mask the biomarker variability associated with disease progression. Given the heterogeneity of periodontitis, biomarker expression may vary according to the degree of tissue destruction and disease progression [1, 3].

Despite these limitations, the present findings indicate that canine periodontal disease is characterized by stage‐dependent immune‐inflammatory and oxidative stress profiles. Gingivitis is predominantly associated with acute inflammatory activation, whereas periodontitis is more closely linked to systemic oxidative stress and IL‐6‐associated chronic inflammatory activity.

From a systems biology perspective, periodontal disease should be considered an integrated host–microbe–immune interaction network rather than isolated biomarker changes. The observed serum–GCF correlations support the potential utility of minimally invasive sampling for monitoring periodontal disease activity in veterinary patients.

## 5. Conclusion

Canine gingivitis and periodontitis exhibit distinct inflammatory and oxidative stress profiles rather than a uniform increase in all biomarkers across the disease stages. Gingivitis is characterized by early immune activation and local inflammatory mediator release, whereas periodontitis is associated with systemic inflammatory and oxidative stress‐related changes, particularly elevated IL‐6 and TOS levels.

The observed serum–GCF associations suggest that local periodontal inflammation is reflected systemically, supporting biomarker profiling as an adjunct to conventional clinical assessment.

Further studies with larger cohorts and longitudinal designs are required to validate these findings and clarify the mechanisms underlying periodontal disease progression.

## Funding

This study was supported with the Project Number TDK‐2025‐3996 within the framework of the Graduate Thesis Project (Doctorate) program of the Scientific Research Projects Coordinator of Ankara University.

## Conflicts of Interest

The authors declare no conflicts of interest.

## Data Availability

The data that support the findings of this study are available upon request from the corresponding author. The data are not publicly available due to privacy or ethical restrictions.
